# Optimization of Effective Minerals on Riboflavin Production by *Bacillus subtilis subsp. subtilis* ATCC 6051 Using Statistical Designs

**Published:** 2018

**Authors:** Marjan Oraei, Seyed Hadi Razavi, Faramarz Khodaiyan

**Affiliations:** Bioprocess Engineering Laboratory (BPEL), Department of Food Science, Engineering and Technology, Faculty of Agricultural Engineering and Technology, University of Tehran, Karaj, Iran

**Keywords:** *Bacillus subtilis* ATCC 6051, Minerals, Riboflavin

## Abstract

**Background:**

Riboflavin (vitamin B_2_) is an essential component of the basic metabolism, and an important nutritional and growth factor in humans, animals, plants and micro-organisms. It has been widely used in the fields of pharmaceuticals, feed and food additives. The industrial production of riboflavin mostly relies on the microbial fermentation. Designing an appropriate fermentation medium is of crucial importance to improve the riboflavin production.

**Methods:**

In this study, sequential methodology combining a screening test of minerals by Plackett-Burman (PB) and an optimization test by Central Composite Design (CCD) was applied to enhance riboflavin production by *Bacillus subtilis* ATCC 6051 in shake flasks.

**Results:**

Initially, one-factor-at-a-time approach was applied to evaluate the effect of different carbon sources. The results showed that fructose was significantly most effective on biomass and riboflavin production. After that, 13 minerals [CaCl_2_, CuCl, FeCl_3_, FeSO_4_, AlCl_3_, Na_3_MoO_4_, Co(NO_3_)_2_, NaCl, KH_2_PO_4_, K_2_HPO_4_, MgSO_4_, ZnSO_4_, and MnSO_4_] were studied with the screening test. The results revealed that concentration of MgSO_4_, K_2_HPO_4_, and FeSO_4_ had greater influence on riboflavin production (p< 0.05). A CCD with five factors (concentration of fructose, MgSO_4_, K_2_HPO_4_, FeSO_4_, and yeast extract) at five levels was then used to determine the maximum riboflavin concentration. The optimal concentrations (*g/l*) of these variables determined by Response Surface Methodology (RSM) were fructose, 38.10; MgSO_4_, 0.85; K_2_HPO_4_, 2.27; FeSO_4_, 0.02; and yeast extract, 4.37.

**Conclusion:**

Statistical experimental design offers a practicable approach to the implementation of medium optimization. From an industrial view point, our optimum medium, besides fructose and a small amount of yeast extract, is mainly composed of common and cheap inorganic salts, which are available to the industrial riboflavin production.

## Introduction

Riboflavin (vitamin B_2_) is an important nutritional and growth factor in humans, animals, plants and microorganisms. This water soluble vitamin is an essential component of the basic metabolism, the precursor of oxidation-reduction coenzymes, Flavin Mononucleotide (FMN) and Flavin Adenine Dinucleotide (FAD) ^1,2^. Because of its particular physiological role and disability of animals and humans in riboflavin production, it has been widely used in the fields of feed and food additives and pharmaceuticals ^3,4^.

Industrial production of riboflavin can be achieved by chemical or biological synthesis, but today, because of the advantages of biotechnical processes such as cost effectiveness, reduction in waste and energy requirements, and the use of renewable resources, it has shifted completely from chemical synthesis to microbial fermentation ^5,6^.

Riboflavin is produced by several microorganisms. Among them, three species can produce higher level of riboflavin and are used for industrial riboflavin production involving the fungi *Ashbya gossypii* and *Eremothecium ashbyii*, the yeast *Candida famata*, and the bacteria *Bacillus subtilis* (*B. subtilis*) ^4,7,8^. *Bacillus* species are used for industrial and commercial biotechnical processes, because many of them are Generally Regarded As Safe (GRAS) by the US Food and Drug Administration and *Bacillus* species such as *B. subtilis* grow rather fast which allows short production cycles ^9,10^. The gram-positive bacterium, *B. subtilis*, is the most important commercial producer of riboflavin ^11^.

Many fermentation factors such as pH ^12^, agitation rate ^13,14^, incubation temperature, *etc*., could affect the riboflavin production. The composition of media culture is one of the most important factors in fermentation reactions including riboflavin production. Optimization of the medium is necessary in microbial fermentations to fully exploit the potential of selected microbial strains ^15^. Any *B. subtilis* strain has different nutritional requirements. *B. subtilis* ATCC 6051 is one of the strains of *B. subtilis* that there is no comprehensive research on its riboflavin productivity. In this study, effective minerals and sugars were selected and optimized to improve riboflavin production by *B. subtilis* ATCC 6051. These results were used for further researches conducted by authors.

Designing an appropriate fermentation medium is of crucial importance to improve the riboflavin concentration, yield, volumetric production, and the ease of downstream product separation ^16^. Micro-organisms require minerals for their growth and metabolic product formation. The requirement of minerals varies with the type of organism ^17^. A sequential optimization strategy, based on statistical experimental designs such as Plackett-Burman (PB) ^18^ and response surface methodology ^19^ which can optimize all the affecting parameters collectively, was used to evaluate the effects of different minerals on riboflavin production and enhance the production of riboflavin by *B. subtilis* ATCC 6051 in shake flask cultures. After preliminary experiments of carbon source selection using one-factor-at-a-time approach, the PB design was implemented to screen minerals that significantly influence riboflavin production. PB design proposes a good and fast screening method and mathematically computes the significance of the large numbers of factors in a few experiments, which saves time and cost and maintains convincing information on each media component ^20^. After that, the optimal values of selected media components were determined by RSM based on the central composite design. RSM which involves factorial design and regression analysis, helps for estimation of effective factors and building models to study interaction between the variable factors and select optimum conditions of them for a desirable response ^21^.

## Materials and Methods

### Materials

The media ingredients including glucose, fructose, maltose, arabinose, yeast extract, nutrient agar and mineral salts [CaCl_2_.2H_2_O, CuCl, FeCl_3_.6H_2_O, FeSO_4_. 7H_2_O, AlCl_3_.6H_2_O, Na_3_MoO_4_.2H_2_O, Co(NO_3_)_2_.6H_2_O, NaCl, KH_2_PO_4_, K_2_HPO_4_, MgSO_4_.7H_2_O, ZnSO_4_.7H_2_O, MnSO_4_.4H_2_O] were obtained from Merck Chemical Co. (Darmstadt, Germany). The riboflavin standard was purchased from Sigma-Aldrich (Sigma-Aldrich Co., United States).

### Micro-organism

The strain of bacterium *B. subtilis subsp. subtilis* (ATCC 6051) used in this work was obtained from Iranian Research Organization for Science and Technology (IROST). It was kept on nutrient agar plates. Every month, single colonies were transferred to a fresh plate, incubated for 3 days at 30°*C*, and then maintained under refrigeration at 4°*C*.

### Preparation of inoculum

Single colonies of *B. subtilis* from the nutrient agar plate were transferred into 250 *ml* Erlenmeyer flask containing calculated amounts of medium (glucose or fructose, 20 *g/l*; yeast extract, 5 *g/l*), incubated in an orbital shaking incubator (Stuart S150, UK) at 200 *rpm* and 30°*C*, and after 12 *hr* used as the pre-culture.

### Culture media and growth conditions

For all treatments, 5% (*v/v*) of inoculum were inoculated into 250 *ml* Erlenmeyer flasks containing 30 *ml* medium (according to the experimental designs). Finally, the flasks used to produce riboflavin were incubated in an orbital shaking incubator at 200 *rpm* and 30°*C* for 72 *hr*. More detailed information of the sequential experiments can be found below.

### Pre-experiment of carbon source selection

Glucose, fructose, maltose, and arabinose were used for selection of the best carbon source among them for riboflavin production by *B. subtilis* ATCC 6051. 40 *g/l* of each sugar and 10 *g/l* yeast extract were used as the media components for all treatments. All the experiments were carried out independently in triplicates, and the results were the average of three replicate experiments (Table 1).

**Table 1.** Effect of addition of different sugars on riboflavin production by *B. subtilis*

**Carbon sources**	**Biomass (*g/l*)**	**Riboflavin (*mg/l*)**
**Glucose**	1.41±0.09 ^a^	1.96±0.07 ^a^
**Fructose**	3.05±0.18 ^b^	3.85±0.06 ^b^
**Maltose**	1.77±0.03 ^a^	3.39±0.12 ^c^
**Arabinose**	2.21±0.49 ^c^	1.12±0.11 ^d^

Conditions: carbon source, 40 *g/l*; yeast extract, 10 *g/l*; temperature, 30°*C*; in rotary shaker, 200 *rpm*; cultivation period, 3 days.

The results are the average of three replicate experiments. Values represent as mean±standard deviation.

a–d) Values in the same columns followed by different superscript letters are significantly different (p<0.05).

### Screening test of minerals

The variable components [CaCl_2_.2H_2_O, CuCl, FeCl_3_. 6H_2_O, FeSO_4_.7H_2_O, AlCl_3_.6H_2_O, Na_3_MoO_4_.2H_2_O, Co (NO_3_)_2_.6H_2_O, NaCl, KH_2_PO_4_, K_2_HPO_4_, MgSO_4_.7H_2_O, Zn SO_4_.7H_2_O, MnSO_4_.4H_2_O] according to the experimental designs (Tables 2 and 3) and the constant media components (fructose, 40 *g/l*; yeast extract, 10 *g/l*) were added for all treatments.

**Table 2.** Range of variables (*g/l*) at different coded levels for the Plackett-Burman design

**Variables (X_i_)**	**Components**	**Level of variables (*g/l*)**		

		**−1**	**0**	**+1**
**X_1_**	KH_2_PO_4_	0	3	6
**X_2_**	K_2_HPO_4_	0	5	10
**X_3_**	MgSO_4_	0	0.75	1.50
**X_4_**	ZnSO_4_	0	0.025	0.050
**X_5_**	MnSO_4_	0	0.025	0.050
**X_6_**	NaCl	0	2.5	5.0
**X_7_**	FeSO_4_	0	0.02	0.04
**X_8_**	FeCl_3_	0	0.01	0.02
**X_9_**	CaCl_2_	0	0.2	0.4
**X_10_**	CuCl	0	0.02	0.04
**X_11_**	AlCl_3_	0	0.005	0.010
**X_12_**	Co(NO_3_)_2_	0	0.03	0.06
**X_13_**	Na_3_MoO_4_	0	0.005	0.010

**Table 3.** Coded levels of the variables in PB design: KH_2_PO_4_ (X_1_); K_2_HPO_4_ (X_2_); MgSO_4_ (X_3_); ZnSO4 (X_4_); MnSO_4_ (X_5_); NaCl (X_6_); FeSO4 (X_7_); FeCl3 (X_8_); CaCl2 (X_9_); CuCl (X_10_); AlCl3 (X_11_); Co(NO_3_)_2_ (X_12_); Na_3_MoO_4_ (X_13_)

**Run**	**Design Matrix**													**Experimental results**	

	**X_1_**	**X_2_**	**X_3_**	**X_4_**	**X_5_**	**X_6_**	**X_7_**	**X_8_**	**X_9_**	**X_10_**	**X_11_**	**X_12_**	**X_13_**	**Biomass (*g/l*)**	**Riboflavin (*mg/l*)**
**1**	+1	−1	+1	+1	−1	−1	−1	−1	+1	−1	+1	−1	+1	9.05	1.23
**2**	+1	+1	−1	+1	+1	−1	−1	−1	−1	+1	−1	+1	−1	2.22	1.35
**3**	−1	+1	+1	−1	+1	+1	+1	−1	−1	−1	+1	−1	+1	7.47	4.32
**4**	−1	−1	+1	+1	−1	+1	+1	−1	−1	−1	−1	+1	−1	8.29	0.53
**5**	+1	−1	−1	+1	+1	−1	+1	+1	−1	−1	−1	−1	+1	3.51	1.06
**6**	+1	+1	−1	−1	+1	+1	−1	+1	+1	−1	−1	−1	−1	2.87	1.04
**7**	+1	+1	+1	−1	−1	+1	+1	−1	+1	+1	−1	−1	−1	6.46	3.78
**8**	+1	+1	+1	+1	−1	−1	+1	+1	−1	+1	+1	−1	−1	5.69	3.42
**9**	−1	+1	+1	+1	+1	−1	−1	+1	+1	−1	+1	+1	−1	6.84	1.32
**10**	+1	−1	+1	+1	+1	+1	−1	−1	+1	+1	−1	+1	+1	1.35	0.10
**11**	−1	+1	−1	+1	+1	+1	+1	−1	−1	+1	+1	−1	+1	2.74	0.90
**12**	+1	−1	+1	−1	+1	+1	+1	+1	−1	−1	+1	+1	−1	5.75	2.27
**13**	−1	+1	−1	+1	−1	+1	+1	+1	+1	−1	−1	+1	+1	3.25	2.49
**14**	−1	−1	+1	−1	+1	−1	+1	+1	+1	+1	−1	−1	+1	8.07	1.85
**15**	−1	−1	−1	+1	−1	+1	−1	+1	+1	+1	+1	−1	−1	1.41	0.10
**16**	−1	−1	−1	−1	+1	−1	+1	−1	+1	+1	+1	+1	−1	0.10	0.00
**17**	+1	−1	−1	−1	−1	+1	−1	+1	−1	+1	+1	+1	+1	0.13	0.00
**18**	+1	+1	−1	−1	−1	−1	−1	−1	+1	−1	+1	+1	+1	4.28	0.11
**19**	−1	+1	+1	−1	−1	−1	−1	+1	−1	+1	−1	+1	+1	6.25	1.29
**20**	−1	−1	−1	−1	−1	−1	−1	−1	−1	−1	−1	−1	−1	3.96	0.13
**21**	0	0	0	0	0	0	0	0	0	0	0	0	0	6.10	0.53
**22**	0	0	0	0	0	0	0	0	0	0	0	0	0	5.84	0.51
**23**	0	0	0	0	0	0	0	0	0	0	0	0	0	5.74	0.50
**24**	0	0	0	0	0	0	0	0	0	0	0	0	0	6.47	0.59

### Optimization of media components

The effective minerals (MgSO_4_, K_2_HPO_4_, and Fe SO_4_), fructose, and yeast extract were added to shaking flasks according to the experimental designs (Tables 4 and 5).

**Table 4.** Range of variables at different coded levels for the CCD

**Independent variables (X_i_)**	**Components**	**Levels (g/l)**					

		**−α**	**−1**	**0**	**+1**	**+α**
**X_1_**	Fructose	20.00	33.53	50.00	66.47	80.00
**X_2_**	MgSO_4_	0.00	0.34	0.75	1.16	1.50
**X_3_**	K_2_HPO_4_	0.00	2.25	5.00	7.75	10.00
**X_4_**	FeSO_4_	0.00	0.02	0.05	0.08	0.10
**X_5_**	Yeast extract	2.00	3.80	6.00	8.20	10.00

**Table 5.** Coded levels of the variables in CCD: fructose (X_1_); MgSO_4_ (X_2_); K_2_HPO_4_ (X_3_); FeSO_4_ (X_4_); yeast extract (X_5_)

**Run**	**Design matrix**					**Experimental results**	
	
	**X_1_**	**X_2_**	**X_3_**	**X_4_**	**X_5_**	**Biomass (*g/l*)**	**Riboflavin (*mg/l*)**
**1**	0	0	0	0	+α	5.53	4.48
**2**	−1	−1	+1	+1	+1	6.22	3.30
**3**	0	−α	0	0	0	1.26	3.41
**4**	0	0	0	0	−α	1.82	1.05
**5**	+1	−1	−1	+1	+1	3.97	0.19
**6**	+1	−1	+1	−1	+1	4.62	5.68
**7**	+1	+1	+1	−1	−1	3.53	1.65
**8**	0	0	0	0	0	6.02	9.32
**9**	−1	−1	−1	−1	−1	4.21	13.0
**10**	0	0	0	0	0	5.82	8.98
**11**	0	0	0	0	0	5.75	8.82
**12**	+α	0	0	0	0	4.28	2.15
**13**	−1	+1	+1	+1	−1	3.85	0.12
**14**	0	0	0	0	0	5.63	8.90
**15**	0	0	0	+α	0	4.80	1.84
**16**	0	+α	0	0	0	5.29	4.52
**17**	0	0	0	−α	0	6.31	5.33
**18**	+1	−1	+1	+1	−1	3.49	0.13
**19**	0	0	0	0	0	6.06	9.47
**20**	0	0	−α	0	0	5.01	3.22
**21**	−1	+1	+1	−1	+1	7.52	2.55
**22**	−1	+1	−1	+1	+1	6.10	0.30
**23**	+1	+1	−1	−1	+1	5.77	4.20
**24**	+1	+1	−1	+1	−1	2.68	0.10
**25**	−α	0	0	0	0	4.18	4.40
**26**	0	0	+α	0	0	4.88	6.27

α=1.821.

### Analytical methods

After 72 *hr* of incubation, biomass and riboflavin were measured. Dry Cell Weight (DCW) was determined from a calibration curve of known DCW and the corresponding Optical Density at 600 *nm* (OD_600_) ^11^.

For riboflavin measurements, 3 *ml* of the broth cultures were centrifuged (Hettich MIKRO 220R, Germany) at 4500 *g* for 6 *min* to remove the cells. After that, the absorption of the cell-free supernatant at 444 *nm* (A_444_) was immediately measured (Cecil, UK). Riboflavin concentration was converted using a calibration curve constructed by the pure riboflavin standard ^1,22^.

### Statistical experimental methods

The conventional method has been used for selection of the best sugar as the carbon source for riboflavin production by *B. subtilis* ATCC 6051 is the onefactor-at-a-time approach (Table 1) in which a single factor is varied while fixing all others at a specific level ^23^. A One-way Analysis of Variance (ANOVA) test has been used to compare the means of data results (biomass and riboflavin production) using PAWS Statistics (SPSS Inc., US).

**Figure 1.**
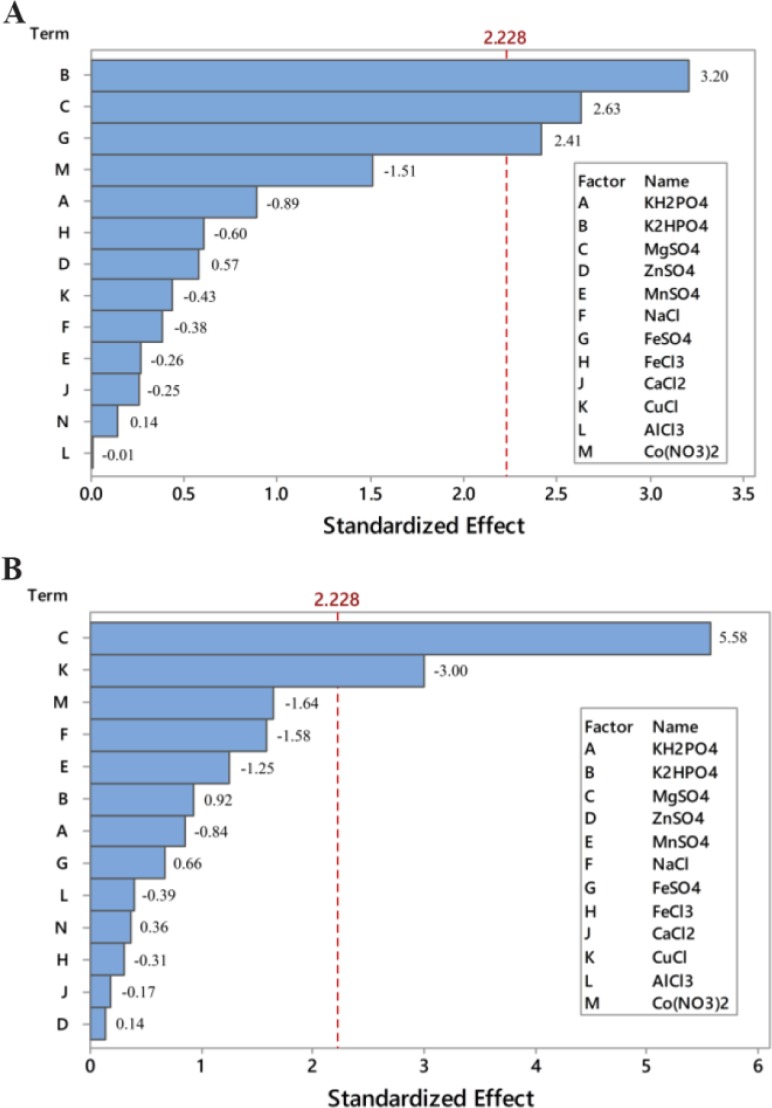
Pareto charts of main effects for Plackett-Burman design on: A) riboflavin production, and B) biomass; (α=0.05).

Screening experiments to select main minerals were performed with 13 factors using a PB design by Minitab 7 (Minitab Inc., US) resulting in 20 experimental runs and four center points (Table 3). The range and the levels of these 13 variables are given in table 2. A pareto chart was used to exclude insignificant factors at an alpha level of 0.05 (Figure 1). The results of the PB design revealed that three out of the 13 factors exerted significant effects on riboflavin production.

RSM based on 5-level-5-factor CCD created by Design-Expert 7 (Stat-Ease Inc., US) was used to optimize the values of the screened minerals, fructose, and yeast extract for enhancing the riboflavin production by *B. subtilis* (Tables 4 and 5). The experimental results of the CCD were fitted with a second-order polynomial equation by a multiple regression technique. The three-dimensional response surface presentations were plotted using Design-Expert 7.

## Results

### Selection of carbon source

Various sugars including glucose, fructose, maltose, and arabinose as the carbon sources were added to the medium cultures. A one-way ANOVA test was used to evaluate the effects of these sugars on riboflavin production by *B. subtilis* ATCC 6051. The highest levels of biomass and riboflavin production were observed in the presence of fructose (Table 1). Hence, fructose was used as the carbon source for further experiments.

### Screening of the significant mineral salts

The primary purpose of screening experiments is to select or screen out the few important main effects from the less important ones ^24^. In this study, a PB design was used to detect the influence of the 13 minerals on biomass and riboflavin production in shake flasks. Table 3 represents the PB experimental design and the corresponding riboflavin production and biomass.

The pareto chart, which has been described as a useful tool for identifying the most important effects ^25^, was applied to determine the significant factors. In this chart, the length of each bar on a standardized pareto chart is proportional to the absolute value of its associated regression coefficient or estimated effect. Figure 1 shows pareto charts for biomass and riboflavin production. These charts show that the concentration of MgSO_4_ is the most effective factor on the biomass and the concentrations of MgSO_4_, K_2_HPO_4_, and FeSO_4_ are the most effective factors on riboflavin production by *B. subtilis* ATCC 6051 (p<0.05). The effects of these three minerals were all positive. Hence, only these three minerals were used for further optimization experiments. In terms of K_2_HPO_4_, it offers the phosphorus source for energy substance, which is important to cell growth and product formation ^3^. Riboflavin is converted into catalytically active cofactors (FAD and FMN) by the actions of riboflavin kinase which contains a magnesium binding site ^26^.

Sabry *et al* ^27^ also found that K_2_HPO_4_ and MgSO_4_ had significant influences on enhancing riboflavin production by *Candida guilliermondii*. Furthermore, figure 1A indicates that the effect of the concentration of CuCl on biomass was significant but negative. Therefore, it was not used for further experiments.

Wu *et al* ^3^ used PB design to screen medium components for recombinant *B. subtilis* RH44. Among 15 variables tested, glucose, NaNO_3_, K_2_HPO_4_, ZnSO_4_, and MnCl_2_ were identified as the most significant factors for riboflavin production. The optimal values of these five variables were determined by RSM. Although the effects of MgSO_4_, sodium citrate, FeCl_2_, and yeast extract were insignificant, they were chosen at their high levels according to the positive effects.

Li *et al* ^28^ screened 11 medium components for riboflavin production of recombinant *B. subtilis* X42 by PB design. Among the tested variables, glucose, yeast powder, MgSO_4_, urea, CuCl_2_ and MnCl_2_ had the greatest impacts on production of riboflavin. But the positive or negative effects of significant factors were not determined.

### Optimization of riboflavin production

A central composite design for the five factors (concentrations of fructose, MgSO_4_, K_2_HPO_4_, FeSO_4_, and yeast extract), each at five levels and five replicates at the center points (to account for pure internal error), was applied for optimizing riboflavin production in shake flasks. The range and the levels of the variables are given in table 4. The design matrix for these factors in the optimization runs and the results are noted in table 5.

By applying multiple regression analysis on the experimental data, the following second order polynomial equation was found to describe riboflavin production.

In this quadratic model, *Y* predicted response and *X*_1_, *X*_2_, *X*_3_, *X*_4_, and *X*_5_ are the coded values of fructose, MgSO_4_, K_2_HPO4, FeSO_4_, and yeast extract, respectively. The ANOVA results are given in table 6 to check the adequacy of the suggested model. The ANOVA results indicated that the quadratic regression to produce the second-order model was significant (p= 0.0054). R-Squared (R^2^), a measure of the model’s goodness of fit was 0.89. The adjusted R^2^ value (0.84) also indicated the goodness of fit of the model.

$$\begin{array}{l} Y=8.37-0.21X_{1}+0.38X_{2}+0.01X_{3}-1.11X_{4}+0.43X_{5}+\\ 1.32X_{1}X_{3}+1.42X_{1}X_{5}+1.47X_{3}X_{4}+2.08X_{3}X_{5}+0.98X_{4}X_{5}-\\ 1.21X_{1}^{2}-1.01X_{2}^{2}-0.77X_{3}^{2}-1.12X_{4}^{2}-1.37X_{5}^{2} \end{array}$$

**Table 6.** Analysis of variance for the selected quadratic model

**Source**	**Sum of Squares**	**df**	**Mean Squares**	**F Value**	**p-value Prob>F**	**R-Squared**	**Adj R-Squared**	**Pred R-Squared**
**Model**	282.46	15	18.83	5.35	0.0054	0.89	0.84	0.71

**Figure 2.**
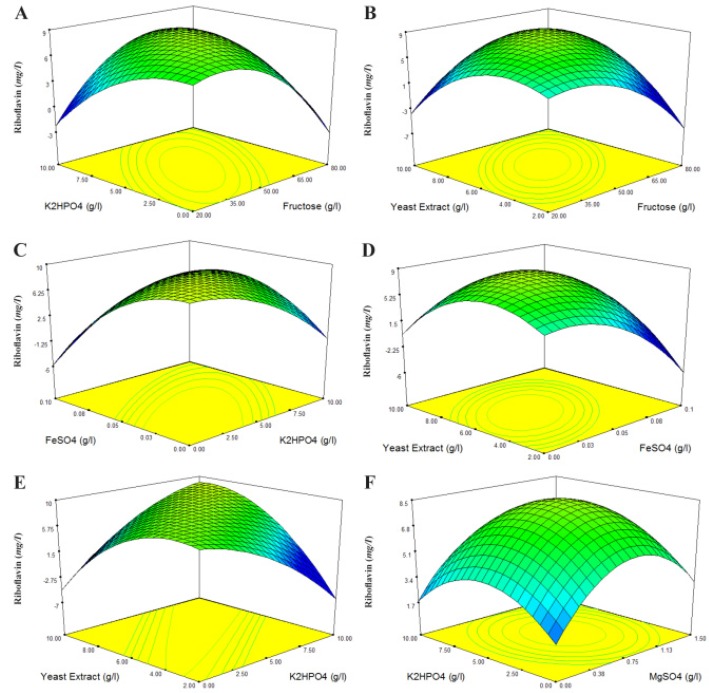
3D Surface plots of the model equation fitted to the data of the central composite design. Interactions of (a) K_2_HPO_4_ and fructose, (b) yeast extract and fructose, (c) FeSO_4_ and K_2_HPO_4_, (d) yeast extract and FeSO_4_, (e) yeast extract and K_2_HPO_4_, (f) K_2_HPO_4_ and MgSO_4_, concentrations at the fixed center values (g/l) of other three remaining factors (fructose, 50; MgSO_4_, 0.75; K_2_HPO_4_, 5; FeSO_4_, 0.05; yeast extract, 6).

The three-dimensional response surface curves were then plotted to understand the interactions of the medium components and find the optimum concentration ranges of components required for maximum riboflavin concentration. Figure 2 is the response surface for variation in riboflavin concentration, as a function of two variables with the other three nutrients being at their constant levels. Figure 2 contains the 3D Surface plots of the model equation fitted to the data of the CCD. Figures 2A–F show the interactions of concentrations of K_2_HPO_4_ and fructose, yeast extract and fructose, FeSO_4_ and K_2_HPO_4_, yeast extract and FeSO_4_, yeast extract and K_2_HPO_4_, and K_2_HPO_4_ and MgSO_4_, respectively, at the center values of other three remaining factors. Figure 2 shows that increasing concentrations of components has a positive influence on maximum riboflavin production until an optimum value is obtained.

## Discussion

The optimum concentrations of fructose, MgSO_4_, K_2_HPO_4_, FeSO_4_, and yeast extract for the highest riboflavin production (12.08 *mg/l*) were 38.10, 0.85, 2.27, 0.02, and 4.37 *g/l*, respectively. The confirmatory experiments were performed in three replicates to validate the optimal point. The riboflavin concentration obtained was 11.73±0.68 *g/l*. It indicates that there is a good correlation between the observed values in experiments and the values predicted by model.

Abdulla *et al* ^29^ found the optimal concentrations (*g/l*) of NaNO_3_ 3, 5; KH_2_PO_4_ 2.5, 1.5; K_2_HPO_4_ 1, 0.5 and MgSO_4_ 0.1, 0.5 without primary screening and only by RSM for riboflavin production by *B. subtilis* ASU8 (KU559874) and *Bacillus tequilensis*, respectively ^30^ reported that the maximum riboflavin production by *Candida sp* LEB 130 was achieved with mineral concentrations of KH_2_PO_4_ 2 *g/l*, MgSO_4_ 1 *g/l* and ZnSO_4_ 0.5 *ml/l* (0.2% solution). Both articles did not use screening test for selection of effective minerals and only optimized the concentration of three or four minerals by RSM.

## Conclusion

Using a sequential optimization strategy (PB design followed by CCD coupled with response surface analysis), the concentration of minerals particularly MgSO_4_, K_2_HPO_4_, and FeSO_4_ were shown to affect the production of riboflavin by *B. subtilis* ATCC 6051 (p<0.05). The response surface analysis of the CCD results indicates that the optimum medium concentrations (*g/l*) for the highest riboflavin production were fructose, 38.10; MgSO_4_, 0.85; K_2_HPO_4_. 2.27; FeSO_4_, 0.02; and yeast extract, 4.37. The maximum riboflavin production (11.73±0.68 *g/l*) was demonstrated by verification experiments of the optimal medium in 72 *hr* shake flask fermentation.

In short, the improvement of riboflavin production observed during this study demonstrated the potential of this strain and provided relevant data on these preliminary tests for further investigation of the strain and the culture medium composition. Furthermore, even though this wild-type *B. subtilis* ATCC 6051 produces lower vitamin concentrations than commercial strains, it may be an important candidate for genetic modification to improve riboflavin production or using waste products such as molasses for more economical production.

## References

[1] Liu S Kang P Cui Z Wang Z Chen T Increased riboflavin production by knockout of 6-phosphofructokinase I and blocking the Entner-Doudoroff pathway in Escherichia coli. Biotechnol Lett 2016;38(8):1307–1314. 27071937 10.1007/s10529-016-2104-5

[2] Dmytruk K Lyzak O Yatsyshyn V Kluz M Sibirny V Puchalski C Construction and fed-batch cultivation of Candida famata with enhanced riboflavin production. J Biotechnol 2014;172:11–17. 24361297 10.1016/j.jbiotec.2013.12.005

[3] Wu QL Chen T Gan Y Chen X Zhao XM Optimization of riboflavin production by recombinant Bacillus subtilis RH44 using statistical designs. Appl Microbiol Biotechnol 2007;76(4):783–794. 17576552 10.1007/s00253-007-1049-y

[4] Stahmann KP Revuelta JL Seulberger H Three biotechnical processes using Ashbya gossypii, Candida famata, or Bacillus subtilis compete with chemical riboflavin production. Appl Microbiol Biotechnol 2000;53(5):509–516. 10855708 10.1007/s002530051649

[5] Kato T Park EY Riboflavin production by Ashbya gossypii. Biotechnol Lett 2012;34(4):611–618. 22187081 10.1007/s10529-011-0833-z

[6] Lim SH Ming H Park EY Choi JS Improvement of riboflavin production using mineral support in the culture of Ashbya gossypii. Food Technol Biotechnol 2003;41 (2):137–144.

[7] Birkenmeier M Neumann S Röder T Kinetic modeling of riboflavin biosynthesis in Bacillus subtilis under production conditions. Biotechnol Lett 2014;36(5):919–928. 24442413 10.1007/s10529-013-1435-8

[8] Burgess CM Smid EJ van Sinderen D Bacterial vitamin B2, B11 and B12 overproduction: An overview. Int J Food Microbiol 2009;133(1–2):1–7. 19467724 10.1016/j.ijfoodmicro.2009.04.012

[9] Knorr B Schlieker H Hohmann H-P Weuster-Botz D Scale-down and parallel operation of the riboflavin production process with Bacillus subtilis. Biochem Eng J 2007;33(3):263–274.

[10] Kabisch J Thürmer A Hübel T Popper L Daniel R Schweder T Characterization and optimization of Bacillus subtilis ATCC 6051 as an expression host. J Biotechnol 2013;163(2):97–104. 22789474 10.1016/j.jbiotec.2012.06.034

[11] Zhu Y Chen X Chen T Shi S Zhao X Over-expression of glucose dehydrogenase improves cell growth and riboflavin production in Bacillus subtilis. Biotechnol Lett 2006;28(20):1667–1672. 16912926 10.1007/s10529-006-9143-2

[12] Kolonne S Seviour R McDougall B Effect of pH on exocellular riboflavin production by Eremothecium ashbyii. Biotechnol Lett 1994;16(1):79–84.

[13] Ozbas T Kutsal T Riboflavin production by Eremothecium ashbyii in a batch stirred tank fermenter. Biotechnol Lett 1986;8(6):441–444.

[14] Man ZW Rao ZM Cheng YP Yang TW Zhang X Xu MJ Enhanced riboflavin production by recombinant Bacillus subtilis RF1 through the optimization of agitation speed. World J Microbiol Biotechnol 2014;30 (2):661–667. 24068533 10.1007/s11274-013-1492-0

[15] Khodaiyan F Razavi SH Mousavi SM Optimization of canthaxanthin production by Dietzia natronolimnaea HS-1 from cheese whey using statistical experimental methods. Biochem Eng J 2008;40(3):415–422.

[16] Kennedy M Krouse D Strategies for improving fermentation medium performance: a review. J Ind Microbiol Biotech 1999;23(6):456–475.

[17] Sikdar DP Majumdar MK Majumdar SK Effect of minerals on the production of the delta endotoxin by Bacillus thuringiensis subsp.israelensis. Biotechnol Lett 1991;13 (7):511–514.

[18] Plackett RL Burman JP The design of optimum multifactorial experiments. Biometrika 1946;33(4):305–325.

[19] Lee M Lee D Cho J Lee J Kim S Kim SW Optimization of enzymatic biodiesel synthesis using RSM in high pressure carbon dioxide and its scale up. Bioprocess Biosyst Eng 2013;36(6):775–780. 23423556 10.1007/s00449-013-0903-9

[20] Abdel-Fattah YR Saeed HM Gohar YM El-Baz MA Improved production of Pseudomonas aeruginosa uricase by optimization of process parameters through statistical experimental designs. Process Biochem 2005;40(5): 1707–1714.

[21] Dalvand MJ Mohtasebi SS Rafiee S Modeling of electrohydrodynamic drying process using response surface methodology. Food Sci Nutr 2014;2(3):200–209. 24936289 10.1002/fsn3.96 PMC4048605

[22] Lin Z Xu Z Li Y Wang Z Chen T Zhao X Metabolic engineering of Escherichia coli for the production of riboflavin. Microb Cell Fact 2014;13:104. 25027702 10.1186/s12934-014-0104-5 PMC4223517

[23] Kalingan AE Liao CM Influence of type and concentration of flavinogenic factors on production of riboflavin by Eremothecium ashbyii NRRL 1363. Bioresour Technol 2002;82(3):219–224. 11991069 10.1016/s0960-8524(01)00194-8

[24] Karian ZA Dudewicz EJ Handbook of Fitting Statistical Distributions with R. 1st ed USA:CRC Press; 2016 1665 p.

[25] Haaland PD Experimental design in biotechnology. 1 st ed USA:CRC press; 1989 248 p.

[26] Serrano A Frago S Velázquez-Campoy A Medina M Role of key residues at the flavin mononucleotide (FMN): adenylyltransferase catalytic site of the bifunctional riboflavin kinase/flavin adenine dinucleotide (FAD) synthetase from Corynebacterium ammoniagenes. Int J Mol Sci 2012;13(11):14492–14517. 23203077 10.3390/ijms131114492 PMC3509593

[27] Sabry S El-Refai A Gamati S Utllization of oil fraction (solar) for riboflavin production byCandida guilliermondii as influenced by some culture conditions. Biotechnol Lett 1988;10(9):615–618.

[28] Li Z Yin G Chen T Optimization of riboflavin production by recombinant bacillus subtilis X42 using statistical designs. Advanced Materials Research, Vols. 634–638, 2013;1031–1036.

[29] Abdulla MH Khalil Bagy MM Nafady NA Morsy FM Mahmoud GAE Activation of riboflavin production by Bacillus subtilis (KU559874) and Bacillus tequilensis (KU559876). EC Bacteriol Virol Res 2016;2(4):131–150.

[30] Suzuki GT Macedo JA Macedo GA Medium composition influence on Biotin and Riboflavin production by newly isolated Candida sp. Braz J Microbiol 2011; 42(3):1093–1100. 24031727 10.1590/S1517-838220110003000030 PMC3768789

